# Using Patient Portals to Improve Patient Outcomes: Systematic Review

**DOI:** 10.2196/15038

**Published:** 2019-12-19

**Authors:** Hae-Ra Han, Kelly T Gleason, Chun-An Sun, Hailey N Miller, Soo Jin Kang, Sotera Chow, Rachel Anderson, Paul Nagy, Tom Bauer

**Affiliations:** 1 The Johns Hopkins University School of Nursing Baltimore, MD United States; 2 The Johns Hopkins University, Center for Cardiovascular and Chronic Care Baltimore, MD United States; 3 The Johns Hopkins University, Center for Community Innovation and Scholarship Baltimore, MD United States; 4 Daegu University, Department of Nursing Daegu Republic of Korea; 5 The Johns Hopkins University, School of Medicine Baltimore, MD United States; 6 The Johns Hopkins Hospitals and Health System Baltimore, MD United States

**Keywords:** patient portal, intervention study, systematic review

## Abstract

**Background:**

With the advent of electronic health record (EHR) systems, there is increasing attention on the EHR system with regard to its use in facilitating patients to play active roles in their care via secure patient portals. However, there is no systematic review to comprehensively address patient portal interventions and patient outcomes.

**Objective:**

This study aimed to synthesize evidence with regard to the characteristics and psychobehavioral and clinical outcomes of patient portal interventions.

**Methods:**

In November 2018, we conducted searches in 3 electronic databases, including PubMed, EMBASE, and Cumulative Index to Nursing and Allied Health Literature, and a total of 24 articles met the eligibility criteria.

**Results:**

All but 3 studies were conducted in the United States. The types of study designs varied, and samples predominantly involved non-Hispanic white and highly educated patients with sizes ranging from 50 to 22,703. Most of the portal interventions used tailored alerts or educational resources tailored to the patient’s condition. Patient portal interventions lead to improvements in a wide range of psychobehavioral outcomes, such as health knowledge, self-efficacy, decision making, medication adherence, and preventive service use. Effects of patient portal interventions on clinical outcomes including blood pressure, glucose, cholesterol, and weight loss were mixed.

**Conclusions:**

Patient portal interventions were overall effective in improving a few psychological outcomes, medication adherence, and preventive service use. There was insufficient evidence to support the use of patient portals to improve clinical outcomes. Understanding the role of patient portals as an effective intervention strategy is an essential step to encourage patients to be actively engaged in their health care.

## Introduction

### Background

Since the enactment of the Health Information Technology for Economic and Clinical Health Act in 2009, a part of the American Recovery and Reinvestment Act, adoption of electronic health record (EHR) systems by hospitals has steadily increased. According to the 2019 Brief by Office of the National Coordinator for Health Information Technology [1], nearly 86% (9/10) of hospitals in the United States now have at least a basic EHR system (eg, patient demographics, problem lists, medication lists, and discharge summaries) [1]. In addition to growth in EHR adoption overall, hospital adoption of technology with advanced functionality has increased significantly. For example, hospital adoption of comprehensive EHR systems—which include the aforementioned basic functions plus more expanded functions such as computerized provider order entry (eg, laboratory tests, radiology tests, medications, consultation requests, and nursing orders), laboratory and diagnostic test result management, and decision support (eg, drug-drug interactions, clinical reminders, or drug dosing support)—has increased from 1.6% in 2008 to more than a third (40%) of US hospitals in 2015 [2].

An examination of 9 hospitals in the United States with a comprehensive EHR system revealed that the EHR systems facilitated patient safety and quality improvement through the use of checklists, alerts, and predictive tools and electronic prescribing and test ordering that reduce errors and redundancy [3]. Similarly, faster communication and streamlined processes through EHR systems led to improved patient flow and quality of care in outpatient cardiology practices [4] and primary care [4,5], although some exceptions exist. For example, a recent analysis [6] using a large registry of hospitalized patients with heart failure (N=21,222) failed to substantiate any association between EHR use and a set of outcomes including quality of care and 30‐day postdischarge death or readmission. Similarly, a longitudinal observational study [7] involving 4 primary care clinics of 2242 patients with diabetes examined EHR messages sent among team members to pass patient care information and found that more frequent EHR message forwarding in primary care teams was associated with worse patient outcomes and higher medical costs.

Although the existing literature has much emphasis on clinician and system use of EHR, increasingly closer attention is being paid to the EHR system in terms of its use in facilitating patients to play active roles in their care via a portal—a secure Web-based site tied to an EHR that gives patients access to their health records, appointment scheduling, refill requests, or secure messaging with the health care team. For example, a recent state of the science review [8] examined patient experiences with portals. The review found that patients’ interest and ability to use the patient portals was influenced by personal factors, such as age, ethnicity, education level, health literacy, health status, and role as a caregiver, and that provider endorsement was one of the most influential factors impacting patients’ adoption of the patient portal [8]. In a realist review, Otte-Trojel et al [9] noted patient insight into personal health information, activation of information, interpersonal continuity of care, and service convenience as mechanisms of patient outcome improvements in 32 studies of patient portals published since 2003. A total of 2 systematic reviews [10,11] examined the effect of patient portals on clinical care and patient outcomes. Specifically, Ammenwerth et al [10] reviewed 4 controlled trials published between 1990 and 2011 and found quicker decrease in office visit rates and better adherence to treatment in the patient portal group, compared with a control group. They found no significant changes in health outcomes. Goldzweig et al [11] reviewed 46 studies of various designs (eg, randomized, nonrandomized, and qualitative studies) published between 1990 and 2013. They found that evidence was mixed about the effect of portals on health care utilization (eg, emergency room visits and hospitalizations); portal use was associated with improved outcomes for patients with chronic diseases such as diabetes, hypertension, and depression when used in conjunction with case management [11].

### Objective

The field is rapidly evolving; however, none of the previous systematic reviews have comprehensively addressed the goals, types, and scope of the patient portal interventions and how these interventions are linked to patient outcomes. Given the rapid adoption of comprehensive EHR systems involving patient portals, a comprehensive systematic review on patient portal interventions is warranted. This study aimed to critically appraise evidence on the effects of patient portal interventions on clinical and psychobehavioral outcomes of patients. We examined the detailed characteristics of patient portal interventions and relevant patient outcomes. Our review systematically extends previous efforts by providing an understanding of (1) what constitutes patient portal interventions (scope and nature) and (2) how patient portal interventions achieve desired effects.

## Methods

### Review Design and Study Eligibility

We conducted a systematic review of research evidence designed to assess patient portal interventions. Studies were screened to assess their relevance to the purposes of our systematic review. Articles were included in this review if the study was (1) about patient portals, (2) published in the English language, and (3) included patient outcomes (either behavioral or clinical in nature). Studies were excluded if full texts were not available (eg, conference abstracts) because of its limited information addressing patient portal interventions and associated outcomes. Studies with no measured outcomes and quantitative designs were also excluded.

### Search and Selection of Studies

The search was conducted in November 2018. Following consultation with a health science librarian, 3 databases—PubMed, EMBASE, and Cumulative Index to Nursing and Allied Health Literature—were searched. Search terms included the following: “Electronic Health Records” OR “Medical Records” AND electronic* OR computer* OR “electronic medical record” OR “electronic medical records” OR “electronic health record” OR “electronic patient records” OR “electronic patient record” OR “electronic health records” OR “EMR” OR “EPR” OR “EHR” OR “patient portal” AND “Patient Participation” OR “patient involvement” OR “patient engagement” OR “patient empowerment.” A full search strategy with specific terms for each database can be found in Multimedia Appendix 1.

**Figure 1:**
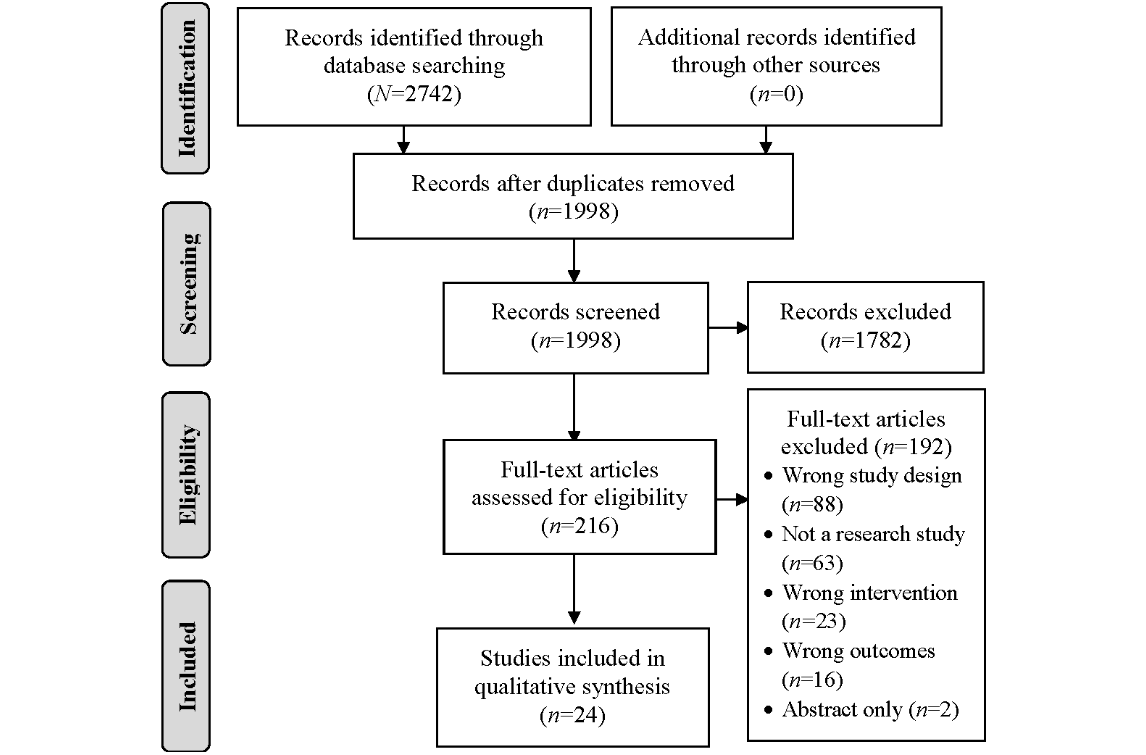
Literature review flowchart.

There were 2742 references that were retrieved from the electronic searches and imported into Covidence software. Of these, 744 duplicates were removed, and 1998 studies were selected for title and abstract screening. A total of 2 reviewers independently conducted an initial screening of titles and abstracts for relevance. In total, 1782 articles were excluded because they were irrelevant. A total of 2 reviewers independently evaluated 216 full-text articles to determine eligibility. Following this, 192 articles were excluded for the following reasons: wrong study design (n=88), not a research study (n=63), wrong intervention (n=23), wrong outcomes (n=16), and abstract only (n=2). All references were screened by 2 independent reviewers. Disagreements were resolved through consensus. A total of 24 articles met the inclusion criteria. Figure 1 provides details of the selection process.

### Data Extraction

Relevant data were extracted by 2 authors using a standardized data extraction form developed by the authors. The following data were extracted from the included studies: first author, publication year, country, study design, study outcomes, measurement, setting, sample sizes, sample demographics, attrition rates, main findings, and patient portal intervention characteristics, including main goal of intervention, type, modality, dose and scope, and patient engagement metrics. An independent research assistant reviewed extracted data to check accuracy. Any discrepancies were resolved through discussions among all research assistants and authors.

### Quality Appraisal

The selected studies were evaluated for quality, based on published quality rating scales to identify strengths and weaknesses in study methodologies and guide the interpretation and assessment of study findings. Specifically, 2 authors rated each study for its quality independently using the Joanna Briggs Institute quality appraisal tool [12]. Each research study’s methodological characteristics were evaluated using the corresponding tool according to study design. A mixed method study [13] was assessed by using both cross-sectional and qualitative checklists. Studies were rated a 0 if they did not identify or include a component of the quality rating and a 1 if they did. Then, the total individual scores (numerator) were added up and divided by the total possible score (denominator) for the respective scale. Studies were rated high, medium, or low quality if they successfully addressed >66.6%, 33.4% to 66.6%, or <33.4% of the components, respectively. Studies were not excluded based on the quality appraisal. Interrater agreement statistics using percent agreement ranged from 66% to 100% (average 88%). Any discrepancies were resolved through team discussions.

## Results

### Quality Ratings: Characterizing the Evidence Base

Tables 1 to 4 show consensual scores of quality assessment. Half of the studies included in this systematic review were of high quality [14-23]. Of the 10 randomized controlled trials (RCTs), 9 were of medium quality [24-32], and 1 was of high quality [33]. Common methodological issues observed in the RCTs had to do with a lack of concealment of allocation to treatment groups, such as nonblinding of participants to treatment assignment [28], nonblinding of those delivering treatment, [24,30,31] or nonblinding of outcome assessors to treatment [24-27,29-31]. Among the quasi-experimental studies, 6 out of 7 [14,16,18,19,21,22] were of high quality, and 1 was of low quality [34]. The low-quality study did not have a control group, did not report if the participants included from the 3 different sites were similar at baseline, did not describe and analyze the incomplete follow-up, and did not report the reliability of the outcome measures. In addition, this study did not have multiple measurements of the outcome both pre- and postexposure to intervention. Of the 6 cohort studies, 4 [15,17,20,23] were of high quality, whereas the remaining 2 [35,36] were of medium quality. These specific studies were of lower rating because of not identifying potential confounding variables or strategies to deal with the confounding variables. The mixed method study [13] was of high quality for its quantitative and cross-sectional methods and of low quality for its qualitative component.

**Table 1:** Study quality ratings for randomized controlled trials.

Items	Studies reviewed									
	Capozza et al, 2015 [24]	Cintron et al, 2006 [25]	Fonda et al, 2009 [26]	Grant et al, 2008 [27]	Krist et al, 2012 [28]	Roach et al, 2010 [29]	Ryu et al, 2017 [30]	Smallwood et al, 2017 [31]	Tang et al, 2013 [33]	Wagner et al, 2012 [32]
Was true randomization used for assignment of participants to treatment groups?	1	1	0	0	0	0	0	0	1	0
Was allocation to treatment groups concealed?	0	0	0	0	0	0	0	0	0	0
Were treatment groups similar at the baseline?	0	1	1	0	0	1	1	1	0	0
Were participants blind to treatment assignment?	0	0	0	0	0	0	0	1	0	0
Were those delivering treatment blind to treatment assignment?	0	0	0	0	0	0	0	0	0	0
Were outcomes assessors blind to treatment assignment?	0	0	0	0	0	0	0	0	1	0
Were treatment groups treated identically other than the intervention of interest?	1	1	1	1	1	1	1	1	1	1
Was follow-up complete and, if not, were differences between groups in terms of follow-up adequately described/analyzed?	1	1	1	1	1	1	1	1	1	1
Were participants analyzed in the groups to which they were randomized?	1	1	1	1	1	1	1	1	1	1
Were outcomes measured in the same way for treatment groups?	0	1	1	1	1	1	1	1	1	1
Were outcomes measured in a reliable way?	0	0	1	1	1	1	1	0	1	1
Was appropriate statistical analysis used?	1	1	1	1	1	1	1	1	1	1
Was the trial design appropriate in the conduct and analysis of the trial?	1	1	1	1	1	1	1	1	1	1

**Table 2:** Study quality ratings for quasi-experimental study.

Items	Studies reviewed						
	de Jong, 2016 [14]	Delbanco et al, 2012 [34]	Greenwood et al, 2014 [16]	Lee et al, 2017 [18]	Milani et al, 2017 [19]	Toscos et al, 2016 [21]	Weisner et al, 2016 [22]
Is it clear in the study what is the *cause,* and what is the *effect*?	1	1	1	1	1	1	1
Were the participants included in any comparisons similar?	0	0	1	0	1	1	1
Were the participants included in any comparisons receiving similar treatment/care, other than the exposure or intervention of interest?	0	0	1	0	1	1	1
Was there a control group?	1	0	1	1	1	0	1
Were there multiple measurements of the outcome both pre- and postintervention/exposure?	1	0	1	1	1	1	1
Was follow-up complete and, if not, were differences between groups in terms of their follow-up adequately described and analyzed?	1	0	0	1	0	0	1
Were the outcomes of participants included in any comparisons measured in the same way?	1	0	1	0	1	1	1
Were outcomes measured in a reliable way?	1	0	1	1	1	1	1
Was appropriate statistical analysis used?	1	1	1	1	1	1	1

**Table 3:** Study quality ratings for cohort study.

Items	Studies reviewed					
	Dumitrascu et al, 2016 [15]	Griffin et al, 2016 [35]	Henry et al, 2016 [23]	Jhamb et al, 2015 [17]	Pecina et al, 2017 [36]	Saberi et al, 2015 [20]
Were the 2 groups similar and recruited from the same population?	1	0	0	0	0	0
Were the exposures measured similarly to assign people to both exposed and unexposed groups?	1	1	1	1	1	1
Was the exposure measured in a valid and reliable way?	1	1	1	1	1	1
Were confounding factors identified?	1	0	1	1	0	1
Were strategies to deal with confounding factors stated?	1	0	1	1	0	1
Were the groups/participants free of the outcome at the start of the study (or at the moment of exposure)?	1	0	0	0	0	1
Were the outcomes measured in a valid and reliable way?	1	0	1	1	1	0
Was the follow-up time reported and sufficient to be long enough for outcomes to occur?	0	0	0	0	0	1
Was follow-up complete, and, if not, were the reasons to loss to follow-up described and explored?	0	0	0	0	0	1
Were strategies to address incomplete follow-up utilized?	0	0	0	0	0	1
Was appropriate statistical analysis used?	1	1	1	1	1	1

**Table 4:** Study quality ratings for mixed method study.

Items		Wade-Vuturdo et al, 2013 [13]
**Quantitative portion**		
	Were the criteria for inclusion in the sample clearly defined?	1
	Were the study subjects and the setting described in detail?	1
	Was the exposure measured in a valid and reliable way?	1
	Were objective, standard criteria used for the measurement of the condition?	1
	Were confounding factors identified?	1
	Were strategies to deal with confounding factors stated?	1
	Were the outcomes measured in a valid and reliable way?	1
	Was appropriate statistical analysis used?	1
**Qualitative portion**		
	Is there congruity between the stated philosophical perspective and the research methodology?	0
	Is there congruity between the research methodology and the research question or objectives?	0
	Is there congruity between the research methodology and the methods used to collect data?	0
	Is there congruity between the research methodology and the representation and analysis of data?	0
	Is there congruity between the research methodology and the interpretation of results?	0
	Is there a statement locating the researcher culturally or theoretically?	0
	Is the influence of the researcher on the research, and vice versa, addressed?	0
	Are participants, and their voices, adequately represented?	1
	Is the research ethical according to current criteria or, for recent studies, is there any evidence of ethical approval by an appropriate body?	1
	Do the conclusions drawn in the research report flow from the analysis, or interpretation, of the data?	1

### Overview of Studies

Multimedia Appendix 2 summarizes the main characteristics of 24 studies included in this review. Of the 24 included studies, 10 [24-33] were RCTs, 7 [14,16,18,19,21,34] were quasi-experimental studies, 1 [13] was a mixed method study using survey and focus groups, 1 [20] was a pre-post cohort study, and the remaining 5 [15,17,23-36] were retrospective cohort studies. Most studies [13,15-17,19-29,31-36] were conducted in the United States. A total of 3 studies [25-27] were published before 2010. A total of 2 studies [15,18] targeted an inpatient population, and all others focused on an outpatient or primary care population. A total of 2 studies [20,34] involved multiple health systems, and all other studies (n=22) were conducted within a single health system. Targeted health conditions included the following: hypertension [17,19,32], depression [22,36], type 2 diabetes [13,16,24,26,27,29,33], HIV [20], osteoporosis or osteopenia [31], coronary artery disease [21], addiction [22], and obesity [30]. Patient outcomes examined included the following: readmission [15], patient knowledge of health information [18,22,25,29,31,33], blood pressure (BP) control [17,19,21,32,33], symptoms of depression [33,36], medication refill adherence [20], blood glucose management [13,21,23,24,26,32,33], weight control [21,27,30,32], preventive health service utilization (eg, cervical, colorectal, and breast cancer screening) [16,23,28,33], and cholesterol control [16,21,30,32,33].

### Characteristics of Patient Portal Intervention

Multimedia Appendix 3 describes the detailed characteristics of patient portal interventions included in the review. The most common patient portal intervention studied was an education tool, available through the portal, tailored to the patient’s condition to provide customized education [14,18,21,23-33]. Another common patient portal intervention was a tailored alert for chronic condition management [16,17,19,24,30], medication refill [14,20,34], or preventive services [23,28] delivered through the patient portal’s secure messaging to the patient. Patient portal activation and use itself [15,21,23,28,32] and, in particular, the use of secure messaging [13,16,20,21,26,32,36], were examined in 12 studies. Primary care providers took part in delivering the intervention in 4 studies [24,26,30], and pharmacists took part in delivering the intervention in 2 of the studies [14,19]. In most studies [13,15-17,19,36], the intervention was a function through the patient portal and without an individual clinician or administrator manually delivering the intervention.

### Effectiveness of Patient Portal Interventions

#### Psychological and Behavioral Outcomes

Effects of patient portal interventions were tested in relation to a variety of psychological (eg, health knowledge, decision making, patient activation, and self-efficacy) and behavioral (eg, adherence and preventive service use) outcomes. Specifically, patient portal interventions were associated with a significant increase in patient knowledge of a health condition or topic in 4 studies [18,25,29,31]. Each of the 4 studies used patient report and a nonstandardized instrument to assess patient knowledge. Similarly, in a pilot RCT [31], patients in the intervention group reported significantly lower conflict in making decisions (measured by the Decisional Conflict Scale) and significantly higher preparation for making decisions (measured by the Preparation for Decision Making Scale). In contrast, 3 quasi-experimental studies reported no significant difference in patient activation [21,22] or patient-reported achievement of behavioral goals (eg, taking medications, healthy eating, being active, monitoring, taking medications, problem-solving, reducing risks, and healthy coping) [16] across the intervention and control group. One of the quasi-experimental studies that did not find a significant difference in patient activation [22] did find that participants in the intervention group were more likely to talk to their health providers about the health topic covered in the intervention. Finally, a quasi-experimental study [14] investigating the impact of the portal’s secure messaging feature reported significantly higher self-efficacy (measured using the Diabetes Management Self-Efficacy Scale) and reports of a collaborative relationship (measured by a self-developed questionnaire) at 26 weeks.

The effects of patient portal interventions on behavioral outcomes were consistently positive. In a cohort study comparing portal users with non–portal users [20], portal users had significantly higher medical refill adherence. Similarly, a quasi-experimental study [34] investigating the impact of the *OpenNotes* feature of the patient portal reported proportionately higher medication adherence measured by patient report and analyzed with summary statistics. A retrospective cohort study [23] and an RCT [28] found that patient portal users were significantly more likely to engage in preventive health care including breast and colorectal cancer screening and Pap smear tests.

#### Clinical Outcomes

A total of 10 studies included in the review reported on clinical outcomes encompassing BP control [17,19,21,32,33], glycemic control [13,16,21,24,26,32,33], cholesterol control [16,21,30,32,33], and weight loss [30,32,33]. In a retrospective cohort study [17] comparing patient portal users with non–patient portal users, portal adoption was only associated with improved BP control in unadjusted models. A quasi-experimental study [19] found that the patient portal intervention was significantly associated with achieving BP control, compared with the control group. The intervention also included a remote, home-based telemonitoring program in addition to the patient portal [19]. An RCT that focused on a tailored patient portal for patients with uncontrolled diabetes and included BP control as a secondary outcome [33] found no significant differences between the intervention and control groups in BP control. Similarly, a quasi-experimental study [21] and a cluster randomized trial [32] found no significant difference in BP control between the intervention and control groups.

Glycemic control, as measured by hemoglobin A_1c_ (HbA_1c_), significantly improved at 6 months, compared with baseline, but the change at 12 months was nonsignificant in patient portal users compared with no patient portal users in both an RCT [33] and a quasi-experimental study [21]. A quasi-experimental study [16], an RCT [24], and a cluster randomized trial [32] also found no difference in glycemic control between the intervention and control groups. A mixed method study with no comparison group found that patient portal use was significantly associated with lower HbA_1c_ values [13]. In addition, an RCT [26] investigating patient portal use found that only the participants randomized to the patient portal who sustained regular use reported significantly lower diabetes distress (measured by the Problem Areas in Diabetes scale), which, in turn, was significantly linked to lower HbA_1c_.

Effects of additional clinical outcomes including cholesterol and weight control were also mixed. For example, cholesterol control, measured by a low-density lipoprotein (LDL) level, was significantly improved in the intervention group of an RCT [33] but was not significantly improved in the intervention group of 2 quasi-experimental studies [16,21], an RCT [30], or a cluster randomized trial [32]. Finally, an RCT [30] and a cluster randomized trial [32] both reported that participants who received the patient portal intervention experienced significant weight loss. In contrast, an RCT [33] investigating a patient portal intervention tailored to patients with uncontrolled type 2 diabetes reported no significant difference in weight loss among the intervention group.

## Discussion

### Principal Findings

To the best of our knowledge, this is the first systematic review that provides a critical appraisal of patient portal interventions with relevant patient outcomes. Although the patient portal interventions varied in their scope, methodology, and outcomes, evidence generally supported the use of patient portal interventions in improving health knowledge [18,25,29,31] and other psychological outcomes, such as decision making [31] and self-efficacy [14], and behavioral outcomes, such as medication adherence [20,34] and cancer screening [23,28]. Patient portal intervention was not effective in improving patient activation [21,22] or behavioral goal achievement [16]. Of particular note, the positive effects of patient portal interventions on medication adherence and cancer screening were consistent across the studies, regardless of the study design, including cohort study [20,34], quasi-experimental study [34], and RCT [28]. These findings suggest patient portal as a promising strategy to improve certain psychological outcomes and health behaviors via simple interventions such as individually tailored messages [28], registration of patients in the Web-based refill services [20,34], or open notes between the patient and the provider [34]. Nevertheless, these studies [20,28,31,34] included predominantly white, middle-aged, and English-speaking populations in their study samples. In addition, the studies reporting positive behavioral outcomes involved a very large sample size (>2000) for which even a small difference (eg, between-group difference of 2.4% in the proportion of patients up-to-date with cancer screening) [28] would result in a statistical significance. Future research is warranted to include patients with more diverse backgrounds (eg, racial/ethnic minorities, older patients, and individuals with limited English proficiency) and of adequate statistical power for testing of applicability and efficacy of patient portal interventions.

Patient portal interventions, overall, had little effects on clinical outcomes addressed in the studies included in the review. For example, of 5 studies in which BP was included as an outcome, only 1 [19] found improved BP control, whereas the other studies did not [21,33] or failed to identify any significant effect in adjusted models [17]. Similarly, less than half of the 7 studies [13,16,21,24,26,32,33] including glucose control as an outcome had a significant finding but either in a noncontrolled setting with no comparison group [13] or only for a short term (6 months) [21,33]. Effects of cholesterol control were also, overall, insignificant, as only 1 [33] of 5 studies had significant reduction in LDL. The overall lack of significant improvements in the clinical outcomes might be attributable to a number of methodological issues such as short-term follow-up or insufficient power to detect changes in outcomes [13,24,26,30]. More important, patient engagement with the portal interventions was not evaluated at all in more than one-third of the studies included in the review [15,17,18,20,23,26,28,31] nor was it systematically incorporated in the design and analysis of the portal interventions. As some studies, where discussed, generally indicated positive changes in patient behaviors or clinical outcomes for individuals with sustained engagement with the portal [21,36], future patient portal interventions should be expanded in scope to focus more on strategies to promote active engagement of patients with the portal.

There are methodological issues to be taken into consideration when interpreting the findings in this review. Although attrition ranged from 0% [18,31] to 71% [34], attrition greater than 20% was observed in more than one-third of the studies using a longitudinal study design [14,24,25,28,29,32,34,36]; another one-third did not report the number and/or reasons for participant withdrawals or dropouts [17,19,20,23,26,35]. Furthermore, 7 studies [14,16,18,19,21,34] used a quasi-experimental study design and, hence, were subject to threats to internal validity. A lack of concealment was also a common methodological issue noted in more than half of the RCTs [24-31]. Nonblinding of those delivering treatment or outcome assessors is likely to have led to the disclosure of group allocation or response bias, hence, threatening the internal validity of the results. Future studies should address these issues by concealing group assignments and separating data collection from intervention delivery. In addition, for reasons not explained in the studies examined, the studies conducted in the United States also lacked complete racial/ethnic diversity by including predominantly white, highly educated, and highly literate in the study samples [13,15-17,21-28,31-33,35,36], and in some cases, such data were not reported [19,20,23,29,34]. The failure to include participants with diverse backgrounds in the sample of studies conducted in the United States limits the generalizability of the study findings. It is furthermore notable that patient portal intervention modalities included in this review involved a form of text messaging activities most often designed for those with high computer literacy skills [32]. Future studies need to include more diverse populations in the study sample such as nonwhites and individuals with limited English proficiency to account for the rapid increase of the populations and those with limited computer literacy. In addition, Future research needs to expand the nature and scope of the modalities in patient portal interventions beyond simple digital text messaging by using a more interactive way of engaging patients, such as using voice and video modalities.

### Limitations

A number of limitations of this review should be noted. First, it is possible that we did not find all relevant articles in the literature. To avoid this, we conducted an extensive systematic electronic search using a compressive list of Medical Subject Heading terms, after consultation with an experienced health science librarian, in addition to hand searches of references of the identified studies. In addition, we did not include gray literature such as reports from organizations; hence, publication bias may exist. We included only articles written in English; therefore, the findings cannot be generalized to studies published in non-English languages. Finally, the studies included in the review used predominantly non-Hispanic white, highly educated, and highly literate individuals, limiting the generalizability of study results. Therefore, the findings from this review should be interpreted with caution.

### Conclusions

Our review of 24 articles of various study designs shows that patient portal interventions can promote positive psychological outcomes for adults in outpatient [14] or primary care [25,29,31] or those in surgery department [18]; increase medication adherence among patients with HIV [20] or those in primary care [34]; and increase cancer screening among those in outpatient or primary care [28]. We were unable to find sufficient evidence to support patient portal interventions as an effective approach for improving clinical outcomes, as some of the included studies reported positive improvements in BP control [17,19], short-term glycemic control [13,21,33], cholesterol control [33], and weight loss [30], whereas others did not [16,21,24,30,32,33]. Although several methodological biases and weaknesses were noted in reference to the patient portal interventions included in this review, our findings suggest the need for more rigorous and continued evaluations of this approach for a broader range of outcomes and populations.

## Appendix

Multimedia Appendix 1 Search strategies by database.

Multimedia Appendix 2 Study characteristics.

Multimedia Appendix 3 Characteristics of patient portal interventions.

## Abbreviations

BP: blood pressure
EHR: electronic health record
HbA_1c_: hemoglobin A_1c_
LDL: low-density lipoprotein
RCT: randomized controlled trial
