# Supplementary material for: Using Patient Portals to Improve Patient Outcomes: Systematic Review
Source: JMIR Hum Factors. 2019 Dec 19;6(4):e15038. doi: 10.2196/15038 (PMC6940868; doi:10.2196/15038)
Supplement: Multimedia Appendix 1 [file humanfactors_v6i4e15038_app1.docx]

**Appendix 1.**

# Search Strategies by Database

## PubMed

((("Electronic Health Records"[Mesh]) OR ("Medical Records"[Mesh] AND (electronic* [tw] OR computer* [tw])) OR "electronic medical record" OR "electronic medical records" OR "electronic health record" OR "electronic patient records" OR "electronic patient record" OR "electronic health records" OR "emr" OR "epr" OR "ehr" OR "patient portal")) AND ("Patient Participation"[Mesh] OR "patient involvement" OR "patient engagement" OR "patient empowerment")

## Embase

('patient engagement'/exp OR 'patient participation'/exp OR ((patient NEAR/3 (involve* OR engage* OR participat*)):ti,ab)) AND ('electronic patient record'/exp OR 'electronic health record'/exp OR 'electronic medical record'/exp OR ('medical record'/exp AND (electronic* OR computer*)) OR ((electronic NEAR/3 ('patient record' OR 'patient records' OR 'medical record' OR 'medical records' OR 'health record' OR 'health records')):ti,ab) OR 'patient portal')

## CINAHL

((MH "Electronic Health Records") OR (MH "Patient Record Systems")) OR ((MH "Medical Records+") AND ( electronic* OR computer* )) OR (( (electronic N3 ("patient record" OR "patient records" OR "medical record" OR "medical records" OR "health record" OR "health records")) ) OR ( EPR OR EMR OR EHR ) OR "patient portal") AND (MH "Consumer Participation") OR ( patient* N3 (involve* OR participat* OR engag*))
