# Supplementary material for: Using Patient Portals to Improve Patient Outcomes: Systematic Review
Source: JMIR Hum Factors. 2019 Dec 19;6(4):e15038. doi: 10.2196/15038 (PMC6940868; doi:10.2196/15038)
Supplement: Multimedia Appendix 2 [file humanfactors_v6i4e15038_app2.docx]

Appendix 2. Study characteristics.

| Author (year), country and reference | Design | Setting | Total (N) and sample (n) numbers and attrition rate | Study period and data points | Sample demographics | Study outcomes and measurement | Main findings | Quality rating |
| --- | --- | --- | --- | --- | --- | --- | --- | --- |
| Capozza et al (2015), United States [23] | 2-arm RCT^a^ | 18 primary care clinics | N=156 patients with type 2 diabetes—intervention=104 and control=52; attrition=24.4% | No study period reported; baseline and 90 and 180 days | Mean age (year)—intervention=52.0 and usual=54; gender (female)—intervention=60% and usual care=63%; race/ethnicity (white)—intervention=76% and usual care=60%; education—not reported | HbA_1C_^b^, patient satisfaction; Client Satisfaction Questionnaire–8 | No statistically significant differences in HbA_1C_ between the 2 groups at 90 and 180 days and satisfaction with the portal intervention was high (27.7/32) with 94% of patients recommending this program to other patients. | Medium |
| Cintron et al (2006), United States [24] | 2-arm RCT | 1 primary care practice | N=912 older (≥50 years) adults—intervention n=430 and control n=482; attrition—overall=51.3%, intervention=55.1%, and control=47.9% | No study period reported; baseline and 6 months | Mean age (years)—not reported; intervention—aged ≥65 years=26%, aged 51-64 years=74%; gender (female)=52%; race/ethnicity (white)=80%; education—not reported | Completion and discussion of HCP^c^, a legal form of advance directives; 4-item HCP survey | The intervention did not significantly affect the outcomes at 6 months: 1% (n=4) of the intervention and 1% (n=6) of the control participants completed HCP and only 1 intervention participant had discussion about HCP; but intervention group was more likely to report knowledge of HCPs (adjusted risk ratio 1.07; 95% CI 1.01-1.14). | Medium |
| de Jong et al (2016)), Netherlands [14] | Quasi-experimental study | 2 outpatient pharmacies | N=152 polypharmacy (>5 medications) patients—intervention=78 and control=74; attrition—overall=42.1%, intervention= 23.1%, and control=62.2% | July 2012 to January 2014; baseline and 26 weeks | Mean age (year)—not reported; intervention: aged ≤65 years=55%, aged ≥66 years=45% and nonuser: aged ≤65 years=51.4%, aged ≥66 years=49%; gender (female)—intervention=56% and nonuser= 40%; race—not reported; education (secondary or lower)—intervention=68% and nonuser=50% | Quality of errors in the eMAR^d^, quality of life, self-care, self-efficacy, and collaboration with pharmacist; SF12, Cardiovascular Risk Management questionnaire, Diabetes Management Self-Efficacy Scale, and Collaborative Relationship Scale | No significant difference in the quality of the eMAR (number of eMARs with corrections) between user and nonuser groups at Week 26. No significant change in quality of life. However, self-efficacy in relation to medication use (*P*<.05) and the collaborative relationship with the pharmacist (*P*<.05) increased during the 6-month study period. | High |
| Delbanco et al (2012), United States [33] | Quasi-experimental study | 1 urban medical center, 1 health system of predominantly rural practices, and 1 urban safety-net hospital | N=113 PCPs^e^ and 22,703 patients who used portals; attrition—PCPs= 12.4% and patient=70.6% | Fall 2011; baseline and 12 months | PCPs; Age (year)—mean age not reported; aged 40-49 years=41% (urban medical center), 22% (rural practices), 37% (urban safety-net hospital); gender (female)—49% (urban medical center), 21% (rural practices), 55% (urban safety-net hospital); race—not reported; education: not reported; patients—no data reported | Potential benefits of OpenNotes (eg, feel more in control of the care and take medications better) to patients reported by patients and PCPs | Of 5219 patients who opened at least one note and completed a postintervention survey, 77% to 87% of patients across the 3 sites reported that OpenNotes helped them feel more in control of their care; 60% to 78% of those taking medications reported increased medication adherence. Few PCPs reported longer visits (0%-5%) or more time addressing patients’ questions outside of visits (0%-8%), 3% to 36% of doctors reported changing documentation content, and 0% to 21% reported taking more time writing notes. Overall, 99% of patients wanted OpenNotes to continue, and no doctor elected to stop. | Low |
| Dumitrascu et al (2016), United States [15] | Retrospective propensity score–matched study | Inpatient clinic | N=7538—portal user=1566 and nonuser=5972; attrition N/A^f^ | August 2012 to July 2014; N/A | Age (year), mean (SD)— portal user=58.8 (15.7) and nonuser=62.3 (15.1); gender (female)—portal user=47% and nonuser=52%; race (white)—portal user=91% and nonuser=90%; education—not reported | Inpatient mortality, 30-day readmission, 30-day mortality | No statistically significant difference between the 2 propensity-matched cohorts with respect to inpatient mortality, 30-day readmission, or 30-day mortality | High |
| Fonda et al (2009), United States [25] | 2-arm RCT | Outpatient | N=104 patients with uncontrolled (HbA_1C_>9%) diabetes—intervention=52 and usual care=52; attrition not reported | No study period reported; baseline and 3, 6, 9, and 12 months | Age (year), mean (SD)—mean age=60.9 (10.3), intervention=61.7 (10.1), and usual care=60.0 (10.5); gender (male)=99%; race—white=77% and Hispanic=6%; education (some college and more)=67% | Diabetes distress and HbA_1C_; Problem Areas in Diabetes Scale | Diabetes distress declined over time for both groups (*P*<.05). Sustained users were 6.3 points lower at 12 months compared with initial points (*P*<.05). A lower baseline HbA_1C_ was associated with lower diabetes distress at baseline, and over time, the decrease in HbA_1C_ was associated with further decreases in diabetes distress. Specifically, each 0.1% drop in HbA_1C_ over time was associated with a 0.7 drop in diabetes distress score (*P*=.03) | Medium |
| Grant et al (2008), United States [26] | 2-arm RCT | 11 primary care practices | N=244 patents with uncontrolled (HbA_1C_>7%) diabetes—intervention=126 and control=118; attrition not reported | September 2005 to March 2007; baseline and 12 months | Age (year), mean (SD)—intervention=58.8 (10.1) and control=53.5 (12.3); gender (female)—intervention=43% and control=56%; race/ethnicity (white)—intervention=93% and control=84%; education—not reported | HbA_1C_, BP^g^, and LDL^h^ cholesterol | No significant differences in HbA_1C_, BP, and LDL cholesterol between study arms after 1 year. More patients in the intervention arm had their diabetes treatment regimens adjusted (53% vs 15%; *P*<.05), compared with active controls. | Medium |
| Greenwood et al (2004), United States [16] | Quasi-experimental study | Outpatient | N=150 patients with type 2 diabetes who completed in-person diabetes self-management education—phone=44, secure message=59, and usual care=47; attrition—phone=13.6%, secure message=15.3%, and usual care=17.0% | No study period reported; baseline and 9 months | Mean age in years (SD)=58.5 (11.5); gender (female)=56%; race/ethnicity—white=59%, Asian/Pacific Islander=21.1%, Hispanic=5%, and black=4%; education (some college and more)=70% | HbA_1C_, LDL, and behavioral change; 10-point scale for healthy eating, being active, monitoring, taking medications, problem-solving, reducing risks, and healthy coping | No significant differences among groups in main outcomes (HbA_1C_ and LDL) between baseline and 9-month follow-up. Overall mean change in HbA_1C_ decreased significantly by −0.88% (SD 1.63%; *P*<.05) from baseline to 9 months; mean change in LDL was not significant. No difference in achieving behavioral goals among groups (in-person: 59%, phone: 73%, and secure message: 77%). Overall mean goal achievement improved from 6.2 (SD 2.4) to 7.2 (SD 1.8) from 3 to 9 months (*P*<.05). | High |
| Griffin et al (2016), United States [35] | Retrospective cohort study | 3 outpatient centers | N=2975 patients with acute myocardial infarction, congestive heart failure, or pneumonia at one of the 3 hospitals—nonuser=2480, light user=257, and active user=238); attrition N/A | May to November 2014; baseline and 1-month follow-up | Mean age (year)=66.3; gender (female)=48%; race/ethnicity (white)—66% (82% for active users, 73% for light users, and 64% for nonusers); education—not reported | 30-day readmission | 30-day readmission rates were for 21% active users, 13% for light users, and 15% for nonusers (*P*<.05). For patients who were active users, the odds of being readmitted within 30 days was 66% greater than those who were nonusers (*P*<.05). | Medium |
| Henry et al (2016), United States [34] | Retrospective cohort study | Outpatient | N=838,638—oPAP^i^ user=338,647 and nonuser=449,991); attrition N/A | December 2014 to March 2015; baseline and 90 days | Age (year), mean (SD)—oPAP user=55.1 (16.19) and nonuser= 45.6 (18.92); gender (female)— oPAP user=55% and nonuser= 45%; race/ethnicity—oPAP user: white=55%, Hispanic=22% and nonuser: white=21%, Hispanic=44%; education—not reported | HbA_1C_ testing, pneumonia vaccination, mammogram, Pap smear, and colorectal cancer screening | oPAP users were more likely to complete HbA_1C_ testing (OR^j^ 1.12), mammogram (OR 1.09), Pap smear (OR 1.06), and colorectal cancer screening (OR 1.09) than nonusers after adjusting for age, gender, ethnicity, marital status, BMI^k^, smoking status, health insurance type, membership status, and length of membership. | High |
| Jhamb et al (2015), United States [17] | Retrospective cohort study | 4 university-affiliated outpatient nephrology clinics | N=2803 patients with hypertension—portal user=1098 and nonuser=1705; attrition N/A | January 2010 to December 2012; baseline and 30 months | Age (year), mean (SD)—mean=61 (17), portal user=58 (16), and nonuser=63(18); gender (female)—portal user=50% and nonuser=49%; race (white)—portal user=87% and nonuser=73%; education—not reported | BP control | Portal adoption only correlated with BP control in unadjusted models (*P*<.05); the difference was nonsignificant after adjusting. | High |
| Krist et al (2012), United States [27] | 2-arm RCT; stratified by sex and age | 8 private primary care practices | N=4500—intervention=2250 and usual care=2250; attrition=51.1% | November 2007 to November 2008; baseline and 4 and 16 months | Mean age (year)— not reported; intervention: aged 50-64 years=30%, aged 35-49 years=30%, aged 60-75 years=20% and control: aged 50-64 years=30%, aged 35-49 years=30%, aged 60-75 years=20%; gender (female)—intervention=50% and control=50%; race/ethnicity (white)—intervention=79% and control=80%; education (some college and more)—intervention=65% and control=68% | Percent up-to-date with preventive cancer screening services | At 4 months, delivery of colorectal, breast, and cervical cancer screening increased by 19%, 15%, and 13%, respectively, among users. The proportion of patients up-to-date with all services increased between baseline and 16 months by 4% among intervention patients (from 11% to 15%; *P*<.05) and by 1.5% among control patients (from 11.1% to 12.6%; *P*<.05)—a difference of 2% (*P*<.05). | Medium |
| Lee et al (2017), Korea [18] | Quasi-experimental study | Pediatric plastic and reconstructive surgery | N=102 patients and guardians—CoPHR^l^ portal=50 and CLCP^m^ app=52; attrition=0.0% | Study period not listed; pre-post at the same encounter (30 min interval) | Mean age (year)—CoPHR portal=36 and CLCP app=32; gender (female)—CoPHR portal=66% and CLCP app=71%; race/ethnicity (Korean)=100%; education—not reported | Objective and subjective health knowledge, information satisfaction, overall satisfaction; pre—open-ended and post—3-point scale for information satisfaction and 5-point Likert scales for overall satisfaction | CoPHR group had significant increase in all item scores for objective and subjective health knowledge (health problems, procedures, lab results, appointments, medication, and subjective knowledge; *P*<.05 for all tests). CLCP group also had significant increase in all item scores for objective and subjective knowledge except objective knowledge related to appointments. CLCP group had higher satisfaction with information than CoPHR group (mean 1.54, SD 0.78, vs 1.14, SD 1.00; *P*<.05), but no difference was observed in subjective knowledge and overall satisfaction. | High |
| Milani et al (2017), United States [19] | Quasi-experimental study | Outpatient | N=556 patients with uncontrolled hypertension—intervention=156 and usual care=400 (matched to age, sex, BMI, and BP); attrition not reported | Study period not listed; baseline and 90 days | Age (year), mean (SD)—intervention=68 (10) and usual care=68 (10); gender (female)—intervention=54% and usual care=54%; race/ethnicity—black: intervention=22%, usual care=23% and other (unspecified): intervention=78% and usual care=77%; education— not reported | BP | At 90 days, 71% of patients in intervention achieved BP control, compared with 31% of the usual care group (*P*<.05). Systolic and diastolic BP, mean arterial pressure, and pulse pressure improved significantly in both group (*P*<.05); for intervention group, systolic BP went down from 147/81 mmHg at baseline to 133/76 mmHg at 90 days and for control group, from 147/81 mmHg at baseline to 143/79 mm Hg at 90 days. | High |
| Pecina et al (2017), United States [36] | Retrospective cohort | Tertiary urban clinic | N=1769 patients enrolled in the depression CCM^n^ program—user, that is, those who had any portal contact with the CCM care manager=272 and nonuser=149; attrition—overall=32.7%, portal=24.5%, and nonuser=34.3% | May 2011 to May 2014; baseline and 6 months | Mean age (year)—portal user=38 and nonuser=43; gender (female)—portal user=81% and nonuser=69%; race/ethnicity (white)—portal user=94.8% and nonuser=93.6%; education—not reported | Completion of PHQ-9^o^, remission rate, and dropping out of depression CCM program; PHQ-9 | Patients using the portal had higher rates of PHQ-9 completion (76% vs 66%; *P*<.05) at 6 months but nonsignificant difference in remission rates and dropout of the CCM program between portal users and nonusers | Medium |
| Roach et al (2010), United States [28] | 2-arm cluster RCT | 2 clinics | N=109 primary care patient; attrition—visit 2=33.9%, visit 3=59.6% (ongoing project at the reported time) | Study period not listed; baseline at visit 1, then regularly followed every 3 to 4 months | Mean age (year)—intervention=57 and control=58; gender (male)—intervention=18% and control=23%; race/ethnicity—not reported; education (less than high school)—intervention=33% and control=45% | Number of specific cardiovascular topics (heart attack risks; ways to lower blood glucose, BP, or cholesterol; or ways to stop smoking) discussed | In visit 2, intervention group was more likely to discuss risk for heart attack (72% vs 42%; *P*<.05) and ways to stop smoking (86% vs 0%; *P*<.05) with their providers. There were no statistically significant differences in all 5 topics in visit 3. | Medium |
| Ryu et al (2017), Korea [29] | 2-arm RCT | Outpatient clinic | N=80—intervention=51 and control=29; attrition—overall=15.0%, intervention=13.7%, and control=17.2% | July to September 2016; baseline and 2 and 4 weeks | Age (year), mean (SD)—Intervention=37.5 (8.7) and control=41.3(11.2); gender (female)—intervention=32% and control=2%; race/ethnicity (Korean)=100%; education (some college and more)—intervention=85% and control=84% | Primary: weight and BMI and secondary: total cholesterol, triglycerides, and HDL^p^/LDL cholesterol | Intervention group had significantly more reductions in weight (1.4 kg vs 0.5 kg; *P*<.05), BMI (0.4 kg/m^2^ vs 0.1 kg/m^2^; *P*<.05), and triglyceride (2.6 mmol/L vs 0.7 mmol/L; *P*<.05) than the control group, but not total cholesterol and HDL/LDL cholesterol | Medium |
| Saberi et al (2015), United States [20] | Pre-post cohort study | 2 health systems | N=2467 HIV-positive patients—electronic SMR^q^ users=1453 and nonusers=1014; attrition—users=11.3% and nonusers=not reported | Study period not listed; 12-month period before SMR, 12-month period starting 6 months after initiation of SMR | Mean age (year)—SMR user=49 and nonuser=49; gender (female)—SMR user=6% and nonuser=6%; race/ethnicity—SMR user: white=76%, black=6%, and Latino=10% and nonuser: white=52%, black=21%, and Latino=19%; education—not reported | ART^r^ medication refill adherence measured by records in the pharmacy databases | Percentage of ART refill adherence change was significantly higher in SMR users (−0.11% in users vs −2.05% in nonusers; *P*<.05). A positive *dose-response* relationship between the frequency of SMR use per month and ART adherence (unadjusted mean percentage change of refill adherence <0.5 times=−1.6%, 0.5-1 time=−0.2%, 1-2 times=0.3%, and ≥2 times=1.0%; *P*<.05) was observed. | High |
| Smallwood et al (2017), United States [30] | 2-arm RCT; stratified by diagnosis (osteoporosis vs osteopenia) | 3 primary care clinics | N=50 women (>55 years) with low bone density (T-score <−1)—intervention=29 and control=21; attrition=0.0% | November 2013 to December 2014; baseline, postintervention, and 3 months | Mean age (year)—intervention=68.8 and control=67.8; gender (female)—intervention=100% and control=100%; race/ethnicity (white)—intervention=97% and control=100%; education (some college and more)—intervention=90% and control= 81% | Primary: decision quality (preparation for decision making and decisional conflict) and secondary: treatment decision (via chart review) and shared decision making; Decision Making Scale, Decision Conflict Scale, DECISIONS study 0-4-point scale | Intervention group reported higher preparedness for making decisions about the treatment than controls (mean 68.1 vs 39.0; *P*<.05) postintervention. Decisional conflict scores were significantly lower postintervention for those in intervention, compared with the control (17.8 vs 47.1; *P*<.05) postintervention. At 3 months, intervention arm was significantly more likely to report having made a decision, compared with the control group (83% vs 57 %; *P*<.05). Shared decision-making scores were higher in the intervention group but not significantly different compared with the control group (3.19 vs 2.91) at 3 months. | Medium |
| Tang et al (2013), United States [32] | 2-arm RCT | Outpatient clinic | N=415 patients with uncontrolled type 2 diabetes—intervention=202 and usual care=213; attrition—intervention=7.9% and control=9.4% | March 2008 to December 2009; baseline and 6 and 12 months | Age (year)—intervention=54.0 (10.7) and control=53.5 (10.2); gender (female)—intervention=41% and usual care=39%; race/ethnicity (white)—intervention=60% and usual care=58%; education (some college and more)—intervention=90% and control=86% | Primary: HbA_1C_ and secondary: BP, LDL cholesterol, 10-year Framingham cardiovascular risk score, and health care utilization; Diabetes Treatment Satisfaction Questionnaire, Consumer Assessment of Health care Providers and Systems, and PHQ-9 | Intervention group had significantly better diabetes control than those in the usual care at 6 months (HbA_1C_ adjusted for baseline levels −1.32% vs −0.66%; *P*<.05), not significant at 12 months (−1.14% vs −0.95%); intervention group had significantly better control of LDL cholesterol at 12 months (−6.1 mg/dl vs 0.0 mg/dl; *P*<.05); no differences for total number of physician visits, BP, depression (PHQ-9), weight, Framingham risk scores at 12 months. | High |
| Toscos et al (2016), United States [21] | Quasi-experimental | Outpatient cardiology clinic | N=200 patients with coronary artery disease; attrition=13.5% | Study period not listed; baseline and 6 and 12 months | Mean age (year)— not reported, but >90% were >55 years; gender (female)=28%; race/ethnicity (white)=98%; education (more than high school)=98% | BP, LDL, BMI, HbA_1C_, patient activation, self-efficacy, and perceived health status; Patient Activation Measure | Systolic and diastolic BP, and LDL decreased from baseline to 12 months but not statistically significant; BMI remained unchanged. Mean HbA_1C_ decreased significantly from 6.25% at baseline to 6.09% at 6 months (*P*<.05) but not at 12 months. A significant improvement in HbA_1C_ at 6 months was shown among active users (−0.19%; *P*<.05) and super users (−0.19%; *P*<.05) but no change for the low users | High |
| Wade-Vuturo et al (2013), United States [13] | Mixed method study (survey and focus group) | Academic medical center | N=54 with type 2 diabetes—both focus group and survey=39 and survey only=15; attrition N/A | Study period not listed; N/A | Age (year), mean (SD)=57.1 (8.4); gender (female)=64.8%; race/ethnicity—white=76% and black=20%; education (average years)=15.2 | HbA_1C_ | Greater self-reported secure messaging use (ie, ≥4 on a scale from 1=never to 6=very often) to schedule an appointment was associated with lower HbA_1C_ values (rho=−0.26; *P*<.05); participant age, gender, race, income, or education level were not associated with using secure messaging to schedule an appointment. | High (cross-sectional); low (focus groups) |
| Wagner et al (2012), United States [31] | Cluster RCT | Outpatient clinic at a tertiary academic medical center | N=446 patients receiving addiction treatment—intervention=194 and nonuser=25; attrition—intervention=33.0% and nonuser=24.2% | Study period not listed; baseline and 3, 6, 9, and 12 months | Age (year), mean (SD)—intervention=54.8 (11.9) and control=54.8 (12.9); gender (female)— intervention=75% and control=66%; race/ethnicity—intervention: white=50%, black=45% and control: white=50%, black=47%; education— not reported but highly literate as measured by Rapid Estimate of Adult Literacy in Medicine (range 0-66) with mean (SD) for intervention=62.7(6.7) and control=60.8 (11.6) | BP, BMI, waist circumference, fasting glucose, triglycerides, HDL, LDL, patient empowerment, and patient perception of practice quality; Patient Empowerment Scale, Patient assessment of Chronic Illness Care, and Consumer Assessment of Health care Providers and Systems | Statistically significant group differences were observed for weight (intervention 213.0 lbs vs control 209.3 lbs; *P*<.05), BMI (34.3 vs 33.8; *P*<.05), waist circumference (108 cm vs 107 cm; *P*<.05), and patient perceptions of quality measures in global doctor rating (9.39 vs 9.43; *P*<.05), in provider communication composite (5.68 vs 5.77; *P*<.05), and in office staff composite (5.4 vs 5.6; *P*<.05), all favoring the control arm. Patient empowerment was higher for the intervention arm (41.2 vs 40.1; *P*<.05). Frequency of portal use was associated with fasting glucose, where no use group had a mean change of −13.70 mg/dl, low use group −6.43 mg/dl, medium use group −10.88 mg/dl, and high use group −9.54 mg/dl (*P*<.05). | Medium |
| Weisner et al (2016), United States [22] | Quasi-experimental | Addiction treatment center | N=503 patients receiving addiction treatment—intervention=252 and control=251; attrition—intervention=10.7% and control=8.4% | April 2011 to October 2013; baseline and 6 months | Mean age (year)—not reported; gender (female)—intervention=30% and control=32%; race/ethnicity—intervention: white=62%, Hispanic=19%, black=7%, Asian=6%, and Control: white=60%, Hispanic=21%, black= 8%, Asian=7%; education (some college and more)—intervention=62% and control=58% | Patient activation, having talked with PCP about alcohol and other drug problems, alcohol and drug abstinence, and depression; Patient Activation Measure and PHQ-9 | More participants in the intervention group had an increase of at least three points on Patient Activation Measure scores compared with baseline, but the difference was not statistically significant (*P*=.14). Intervention group had twice the odds of having talked with their PCP about alcohol and other drug problems (OR 2.3; *P*<.05). No significant difference was observed in alcohol and drug abstinence rates at 6 months (71% for intervention vs 67% for usual care). Also, no significant differences for moderate to severe depression between groups were observed. | High |

^a^RCT: randomized controlled trial.

^b^HbA_1C_: hemoglobin A1C.

^c^HCP: health care proxy.

^d^eMAR: electronic medication record.

^e^PCP: primary care physician.

^f^N/A: not applicable.

^g^BP: blood pressure.

^h^LDL: low-density lipoprotein.

^i^oPAP: Online Personal Action Plan.

^j^OR: odds ratio.

^k^BMI: body mass index.

^l^CoPHR: coproduced personal health record.

^m^CLCP: cleft lip and palate.

^n^CCM: collaborative care management.

^o^PHQ-9: Patient Health Questionnaire–9.

^p^HDL: high-density lipoprotein.

^q^SMR: shared medical record.

^r^ART: antiretroviral therapy.
