# Supplementary material for: Using Patient Portals to Improve Patient Outcomes: Systematic Review
Source: JMIR Hum Factors. 2019 Dec 19;6(4):e15038. doi: 10.2196/15038 (PMC6940868; doi:10.2196/15038)
Supplement: Multimedia Appendix 3 [file humanfactors_v6i4e15038_app3.docx]

Appendix 3. Characteristics of patient portal interventions

| Author (year), country and reference | Main goal of the study | Type, modality, dose, and scope of patient portal intervention | Comparison or control | Metrics for patient engagement with portal and evaluation results |
| --- | --- | --- | --- | --- |
| Capozza et al (2015), United States [23] | To test the impact of text messaging program (Care4Life) on glycemic control | Daily 1 to 7 text messages (5th grade reading level) were sent via a portal to a patient’s mobile for 6 months. The patients controlled the type and frequency of the message. They allowed to view their EHR^a^ and enter self-monitoring results and then set for reminding for self-care monitoring (BP^b^ and glucose) | Usual care (no text message) | Number of patient log-in, frequency rate of patient's response according to the core message (eg, medication and exercise,); 29% of the participants had frequent engagement (texting responses >3 times/week) over 90 days |
| Cintron et al (2006), United States [24] | To examine the effect of patient-directed electronic messages intervention on HCP^c^ | Hyperlinked the HCP message reminder at baseline via the Web portal (PatientSite) to patients and repeated at 4 and 8 weeks; patients accessed, completed, and discussed their HCP with a physician for 6 months | Control group (no electronic message) | Number of participants who opened the message; 62% of the participants opened the message |
| de Jong et al (2016), Netherlands [14] | To investigate the effects of patient participation through the use of a patient communication tool (eMAR-PCT^d^) | Patients were invited to check their eMAR after every change in prescription and notify their pharmacists when corrections needed to be made for 26 weeks | Nonuser (no eMAR-PCT) | Number of logins to the eMAR-PCT and the number of emails between pharmacist and patient; 24% logged into the portal once, and 40% viewed it 2 to 5 times during the 6 months, and 18% communicated asynchronously with the pharmacist using email |
| Delbanco et al (2012), United States [33] | To evaluate the effect of OpenNotes on doctors and patients of facilitating patient access to visit notes over secure internet portals | 2 weeks before a next scheduled visit to their PCP^e^; patients received a secured email message to read *OpenNotes* (previsit activation) and could visit it for 12 months | N/A^f^ | Portal use: visit and open OpenNotes; 11,155 of 13,564 patients with visit notes available opened at least one note (84% at urban medical center, 82% at predominantly rural, and 47% at urban safety-net hospital) |
| Dumitrascu et al (2016), United States [15] | To investigate whether the use of a patient portal during hospitalization is associated with improvement in mortality and readmission outcomes | Patients accessed their lab reports, admission notes, consultation reports, operative notes, discharge summaries, radiology, and pathology reports via Web-based portal and mobile app, except daily progress notes | No portal user (could not access one’s medical record in hospitalization) | In-hospital patient portal use; none reported |
| Fonda et al (2009), United States [25] | To examine the effects of IBCM^g^ portal on the relationship between diabetes distress and glycemic control | The portal contained diabetes educational modules and links to other diabetes resources; the home-based BP and glucose data transmitted to the patient portal and reviewed by NP^h^ (certified diabetes educator), and the NP provided care recommendation using treatment algorithms for 12 months; if patients did not contact the portal for 2 week, study coordinator attempted to contact the patients and encourage the usage the portal | Usual care (no IBCM portal intervention) | Log-in history for the patterns of usage (nonuse, early cessation, sustained but irregular use, and sustained and consistent use); none reported |
| Grant et al (2008), United States [26] | To evaluate the impact of a diabetes-specific patient health record on clinical outcomes | Patients were allowed to review one’s medication list and edit inaccuracies and answer questions regarding medication adherence and adverse effects of medication via diabetes-specific patient health record; a *Diabetes Care Plan* based on patients’ responses before a scheduled clinic visit was submitted directly to the EHR used by the patient’s PCP and could also be printed by the patient and brought to the upcoming appointment for 12 months | Active control (health maintenance, family history, and other non–diabetes-specific preventive services instead of diabetes module) | Percent completion of *Diabetes Care Plan*; 65% of the participants in the intervention arm submitted the care plan, and 35% in control arms submitted family history and health maintenance journals |
| Greenwood et al (2004), United States [16] | To determine the feasibility of diabetes self-management support delivered via phone or secure message via EHR and to compare outcomes | After completion of in-person diabetes self-management education, participants self-selected into the following groups: (1) phone follow-up (scheduled phone appointment with a certified diabetes educator for 15-20 min at 3, 6, and 9 months) and (2) secure message via EHR (scheduled messages from a certified diabetes educator electronically through My Health Online at 3, 6, and 9 months) | Usual care (in-person appointment with the diabetes educator in 3-6 months) | Number of attempts and completed contacts; the success rate (number of completed contacts/number of attempts) was 39% (in-person), 46% (phone), and 29% (secure message); phone contact was significantly longer than secure message contact (mean 45.3, SD 28.6 min, vs 17.8, SD 11.8 min; *P*<.05); fewer secure message members (28%) than phone (47%) or usual care members (51%) completed the intervention per protocol (*P*<.05) |
| Griffin et al (2016), United States [35] | To examine whether the level of patient portal is associated with readmission rates | Patients selected parts of their medical record and used SM^h^ to communicate with nonclinical staff or health providers depending on the type of message, used schedule appointments, refilled prescriptions, and managed medical bills via portal | Nonuser (ie, those who declined activation code or failed to log-in within 30 days after first discharge) | Number of logins within 30 days after discharge; average portal user activities during 30 days after discharge were as follows: light users (those who logged <8 times) logged in 3 times, viewed or sent message 9 times, viewed lab reports 14 times, sent medical advice message 0.4 times, and viewed problem list 3 times, medication list 2 times, appointment details 0.4 times, and immunization and allergy 2 times; active users (those who logged in >8 times) logged in 34 times/month, viewed or sent message 109 times, viewed lab reports 76 times, sent medical advice message 6 times, and viewed problem list 9 times, medication list 8 times, appointment details 8 times, and immunization and allergy 7 times |
| Henry et al (2016), United States [34] | To determine whether oPAP^i^ access was associated with higher rates of 5 care gap closure (HbA_1C_^j^ testing, pneumonia vaccination, and 3 cancer screenings—mammogram, Pap smear, and colorectal screening) | Patients got personalized information about prevention, health promotion, and care gaps via oPAP | Non-oPAP user (not register the portal) | None listed; none reported |
| Jhamb et al (2015), United States [17] | To test whether the portal adoption is independently associated with BP control in patients with hypertension | The portal emailed a patient new information or test results and sent message 1 week before scheduled appointments via SM | Nonuser (no access code and failed to log-in into the portal) | Portal usage and log-in; none reported |
| Krist et al (2012), United States [27] | To test the effectiveness of an IPHR^k^ | Patients received personally tailored message about preventive service and its rationale via IPHR; it included links to evidence-based educational material and decision aids; after a patient used the IPHR, the system automatically forwarded a summary to the EHR inbox of the patient’s clinician | Control group (no IPHR) | Percentage of patients who were mailed an invitation to visit the IPHR, established an account, and received prevention recommendations; none reported |
| Lee et al (2017), Korea [18] | To confirm the effectiveness of an app (CLCP^l^) or a patient portal (CoPHR^m^) via the comparison of users' knowledge, information need satisfaction, and overall satisfaction | An app (CLCP) targeting cleft lip/palate; participants used the service for about 30 min after the consent and pretest | CoPHR: a generic portal showing an overview of his/her health problems, vital signs, height, weight, medication, and appointments | None listed; none reported |
| Milani et al (2017), United States [19] | To evaluate the effectiveness of a remote, home-based telemonitoring program on BP control in patients with uncontrolled hypertension | Patients uploaded BP reading wirelessly once a week; medication management and lifestyle recommendations via a clinical pharmacist and a health coach; tailored follow-up included the following: (1) those with medication adherence issues were provided educational materials, pill reminder apps, and resources such as custom videos and downloadable handouts, as well as a simplified medication regimen when possible; (2) patients with positive depression or obstructive sleep apnea got referred to physicians or specialist; and (3) those with high sodium level received education through email with video education and other educational resources on diet | Usual care (whose physician was not participating in the program) | BP submissions per week; for patients who achieved BP control at 90 days (n=111), the average BP submissions was 4.3 (SD 3.8) times per week, and for those who did not achieve BP control at 90 days (n=45), it was 4.3 (SD 3.2) times per week |
| Pecina et al (2017), United States [36] | To evaluate the differences in depression management processes and outcomes for patients who had any portal contact with the depression collaborative care management versus those who did not | A patient portal where patients contacted with the depression collaborative care manager; nurse care managers provided support and education utilizing motivational interviewing techniques while monitoring patient's depression via Patient Health Questionnaire–9 | Those who did not use the portal for a depression collaborative care management program | The number of portal contacts with the care managers; 33% low portal contacts (portal contact =1), 42% medium portal contacts (2-10), and 25% high portal contacts (>10) |
| Roach et al (2010), United States [28] | To describe a cardiovascular disease risk communication intervention designed for an urban primary care population, utilizing an EHR system | EHR created personalized risk messages; a tablet-based health promotion tool with multimedia technology was used to offer initial core module at the first encounter and 6 additional modules for more specific contents (eg, glucose control, lipid control, diet and exercise, and BP control) tailored to sex and ethnic background of each patient; patients viewed different multimedia content at each visit | Usual care | None listed; none reported |
| Ryu et al (2017), Korea [29] | To analyze the effectiveness of PHR^n^-based intervention | The activity tracker (Misfit) and the mobile app (MyHealthKeeper) to record daily food intake and activity logs used; these data transmitted and saved on individual EHR server; on the basis of the record, the clinician provided lifestyle management feedback at least one comment (biweekly visit) and whenever they met patients at outpatient clinic for 4 weeks | Control group (conventional care, no tracker and no mobile app; provided lifestyle modification feedback during the 4-week) | Number of feedback comment made; a total of 88 feedback comments (2 visits per patients) or lifestyle health prescription were issued on the PHR modules |
| Saberi et al (2015), United States [20] | To compare antiretroviral therapy refill adherence change between electronic SMR^o^ users and age- and sex-frequency matched nonusers pre- and post-SMR use | SMR users were those who completed the enrollment process to use the Web-based services; used 1 or more of the 7 SMR functions during the first 24 months post SMR rollout and enrolled in the health plan at least twelve months before the date of first SMR use and maintained enrollment for at least eighteen months after the date of first SMR use | Those who did not use SMR | Frequency of SMR use; none reported |
| Smallwood et al (2017), United States [30] | To determine the feasibility and potential efficacy of a patient portal–based osteoporosis DA^p^ | A Web-based DA delivered via an EHR-based patient portal; information about osteoporosis including causes, risk factors, assessment, personalized fracture risk, details about medication and nonprescription treatment, a value elicitation exercise related to the treatment decision, and a medication table | Control site provided Web-based information relevant to aging but not specific to osteoporosis | None listed; none reported |
| Tang et al (2013), United States [32] | To evaluate a Web-based disease management system supporting patients with uncontrolled type 2 diabetes | Intervention included the following: (1) glucometer upload system that transmits home glucometer readings to EHR, (2) diabetes summary status report and a comprehensive patient-specific *dashboard*, (3) nutrition log, (4) insulin record, (5) exercise log, (6) Web-based messaging system for communicating with health care team, (7) NCMs^q^ who provide advice and make protocol-based changes to medications, (8) patient-specific text and video educational *nuggets* dispensed electronically by the NCMs | Usual care | Active participation in measurement (glucose reading upload) and communication; 88% of the intervention participants wirelessly uploaded home glucose readings; more intervention group patients initiated Web-based messages to providers/NCMs (145 vs 81; *P*<.05) |
| Toscos et al (2016), United States [21] | To determine the impact of tethered PHR use on patient activation and intermediate health outcomes (BMI^r^, BP, low-density lipoprotein, HbA_1C_) among patients with coronary artery disease | Each participant was provided with a PHR account with features including personal information, medical history, prescription history, secure 2-way messaging with health care professionals, and educational resources; a daily health diary was incorporated into the PHR (self-entered and transmitted BP, heart rate, blood glucose, weight, and BMI); patients could sign up for daily reminders to complete and submit their diary information | N/A | Frequency of PHR use; there were 9.43 logins per patient between baseline and 6 months, 8.58 between 6 and 12 months; the proportion of patients who used the daily health diary increased from 16% in the first half of the study to 28% in the second half (*P*<.05); at 12 months, there were 18% low users (logged in 1-3 times over the 12-month period), 63% active users (logged in 4-23 times), and 18% super users (>24 times) |
| Wade-Vuturo et al (2013), United States [13] | To understand why patient portal users with type 2 diabetes use SM or not and to explore the relationship between self-reported SM use within a portal and glycemic control | All office visits scheduled through the portal were managed via SM, and patients could send a message to their providers for any other purpose; patient-initiated messages were managed and addressed by clinic groups including a clinic staff member, nurse, or patients’ physician depending on the message content | N/A | Self-reported use of patient portal features; 63% used SM to send a message to the doctor; 36% used SM to request an appointment |
| Wagner et al (2012), United States [31] | To examine the impact of a PHR in patients with hypertension measured by changes in biological outcomes, patient empowerment, patient perception of quality of care, and use of medical services | PHRs included the following: (1) SM; (2) access to educational materials; (3) medication interaction checking; (4) recording and monitoring of health measures (eg, BP); (4) viewing of some EMR data such as problem lists and information on medications, allergies, immunizations; and (5) goal setting and health diaries; at each study visit, researchers met with patients to collect data, answer questions about the PHR, and provide PHR demonstrations as requested | Usual care | PHR use frequency; patients from family medicine were more likely to use the PHR than those from internal medicine (OR^s^ 3.39, 95% CI 1.52-7.55; *P*<.05); nonusers (used during training only) were older than users (58.70 vs 53.99; OR 0.96, 95% CI 0.93-1.00; *P*<.05), had lower self-rated computer skills (5.15 vs 6.48; OR 1.21, 95% CI 1.05-1.39; *P*<.05), and scored lower on the Internet Accessibility Questionnaire measuring ease of access to and familiarity with technology (3.58 vs 4.8; OR 1.24, 95% CI 1.05-1.47; *P*<.05) |
| Weisner et al (2016), United States [22] | To examine the effects of an intervention aiming to link patients receiving addiction treatment with health care | LINKAGE (intervention group) participants received 6 45-min group-based, manual-guided sessions (2 per week) that focused on demonstrating how health care is related to overall health, assessing and engaging with health care, and improving communication with physicians (eg, using secure email, viewing laboratory test results and medical information, and accessing prevention services) | Usual care medical education sessions | Days of patient portal log-in; intervention group showed an increase in the mean number of log-in days (IRR^t^ 1.53; *P*<.05), log-in days for medical advice (IRR 1.55; *P*<.05), messages sent by a health care professional (IRR 1.45; *P*<.05), log-in days for laboratory test result review (IRR: 1.92, *P*<.05), and log-in days for laboratory test information (IRR: 1.89, *P*<.05) |

^a^EHR: electronic health record.

^b^BP: blood pressure.

^c^HCP: health care proxy.

^d^eMAR-PCT: electronic medication administration record-patient communication tool

^e^PCP: primary care physician.

^f^N/A: not applicable.

^g^IBCM: internet-based care management.

^h^SM: secure messaging.

^i^oPAP: Online Personal Action Plan.

^j^HbA_1C_: hemoglobin A1C.

^k^IPHR: interactive preventive health record.

^l^CLCP: cleft lip and palate.

^m^CoPHR: coproduced personal health record.

^n^PHR: personal health record.

^o^SMR: shared medical record.

^p^DA: decision aid.

^q^NCM: nurse care manager.

^r^BMI: body mass index.

^s^OR: odds ratio.

^t^IRR: incidence rate ratio.
